# Sudoscan's Effectiveness in Identifying Chronic Kidney Disease in Patients With Type 2 Diabetes

**DOI:** 10.7759/cureus.60344

**Published:** 2024-05-15

**Authors:** Andra E Nica, Emilia Rusu, Carmen G Dobjanschi, Florin Rusu, Oana A Parliteanu, Ion V Vinereanu, Claudia Sivu, Gabriela Radulian

**Affiliations:** 1 Diabetes and Endocrinology, Carol Davila University of Medicine and Pharmacy, Bucharest, ROU; 2 Urology, Dr. Carol Davila Central Military Emergency University Hospital, Bucharest, ROU; 3 Ambulatory, Marius Nasta Institute of Pneumology, Bucharest, ROU; 4 Nephrology, Carol Davila University of Medicine and Pharmacy, Bucharest, ROU

**Keywords:** diabetes complications, sudomotor function, chronic kidney disease, t2dm, sudoscan

## Abstract

Chronic kidney disease (CKD) represents a significant public health issue, particularly prevalent among patients with type 2 diabetes mellitus (T2DM). CKD occurs in approximately 20% to 40% of adults with diabetes mellitus. Sudoscan potentially detects CKD early, providing a non-invasive and convenient alternative to traditional screening methods that rely on serum creatinine and urine albumin levels. This research involves 271 patients from a single medical center over one year, with all participants providing informed consent. The prevalence of CKD in our group was 26.5% (*n* = 72). This study integrates a comprehensive examination, including anthropometric measurements, biochemical profiles, and Sudoscan's electrochemical skin conductance testing. CKD diagnosis was confirmed via estimated glomerular filtration rate (eGFR) and albumin-to-creatinine ratio (ACR). The aim of this study was to explore the utility of Sudoscan in detecting CKD among patients with T2DM. Statistical analysis reveals moderate correlations between Sudoscan scores and traditional CKD markers like eGFR and albuminuria. It is beneficial in settings where conventional testing is less accessible, suggesting potential for broader CKD screening programs. Key findings suggest that Sudoscan can identify early renal dysfunction with reasonable sensitivity and specificity. Integrating Sudoscan in regular CKD screening could enhance early detection, allowing for timely interventions to prevent progression to end-stage renal disease and reduce healthcare burdens associated with advanced CKD. The results contribute to the ongoing assessment of innovative technologies in managing chronic diseases related to diabetes.

## Introduction

Chronic kidney disease (CKD) represents a significant public health issue, particularly prevalent among patients with type 2 diabetes mellitus (T2DM), where it acts as both a common complication and a critical predictor of morbidity and mortality [1]. CKD occurs in 20% to 40% of adults with diabetes mellitus [2-5]. The early detection and management of CKD in diabetic populations is pivotal for preventing the progression to end-stage renal disease (ESRD) and reducing the associated healthcare burdens [6]. The assessment of kidney function relies on biochemical markers such as serum creatinine levels, the calculation of estimated glomerular filtration rate (eGFR), and the level of albuminuria [7]. Regular laboratory investigations are necessary to detect CKD and its progression, considering the disease's evolution and the lack of specific symptoms until advanced stages. International guidelines recommend annual screening for CKD, through measuring serum creatinine and urine albumin excretion [8]. These methods have some limitations in terms of sensitivity and specificity, especially in the early stages of CKD [9]. Given these aspects, a reliable and non-invasive new technology, suitable for identifying individuals at risk of CKD, could add value to existing screening strategies for CKD in patients with T2DM. Sudoscan, a device that measures electrochemical skin conductance, has proven to be a promising tool for evaluating sudomotor function, often affected in patients with diabetes due to peripheral autonomic neuropathy. This technology indirectly measures small fiber and autonomic nerve activity, which are crucial in the pathophysiology of diabetes and its renal complications [10]. By evaluating the ability of sweat glands to release chloride ions in response to a small electrical stimulus, Sudoscan may offer valuable insights into the development and progression of renal complications associated with diabetes [11,12].

This study aimed to explore the utility of Sudoscan in detecting CKD among patients with T2DM. By focusing on the electrochemical skin conductance analysis, this research seeks to establish a correlation between Sudoscan scores and traditional markers of kidney health, such as eGFR and albuminuria levels. The hypothesis is that Sudoscan's sensitivity to changes in sudomotor function could allow for easier detection of kidney dysfunction compared to conventional methods.

## Materials and methods

This cross-sectional study enrolled 271 patients and was conducted between June 2019 and June 2020 in a single medical center. This study received ethical approval for research (approval number: 2145) from the Ethics Committee of Nicolae Malaxa Clinical Hospital. The data obtained were per the hospital's standard of care for patients diagnosed with T2DM. Patients included in the study provided consent by signing the informed consent form for data collection and its utilization in medical research. All patients were recruited from the Diabetes Department of Nicolae Malaxa Clinical Hospital.

Study population 

The inclusion criteria included patients evaluated in the outpatient department of the hospital during the study period who signed informed consent. We included patients with T2DM and overweight/obese adults aged 18 and older in the study. On the other hand, the exclusion criteria involved patients who did not sign informed consent, those with other forms of diabetes (maturity-onset diabetes of the young, type 1 diabetes, or latent autoimmune diabetes of adults), individuals under 18 years of age, patients with a history of neoplasms within the past five years, patients who underwent lower limb amputations, pregnant women, individuals with stroke-related complications, a history of myocardial infarction, preexisting CKDs predating diabetes diagnosis, and patients with other causes of neuropathy apart from diabetes (vitamin B12 deficiency and chronic ethanol consumption).

We specifically excluded patients with stroke-related complications from the study due to the potential for false positive results in sensitivity tests or Sudoscan. Patients undergoing chemotherapy for neoplastic conditions were excluded due to the risk of developing neuropathy secondary to specific treatment. Patients with a history of lower limb amputations were excluded because Sudoscan testing cannot be performed in such situations.

Examination of patients

We documented information concerning anthropometric measurements, encompassing height, weight, body mass index (BMI), waist circumference, waist-to-hip ratio, blood pressure measurements in both supine and orthostatic positions, heart rate in both supine and orthostatic positions, as well as the smoking status.

Measurement of biochemical parameters

After an eight-hour fast, the following specimens were collected from venous plasma: glycated hemoglobin (HbA1c), serum glucose, urea, serum creatinine, urinary albumin-to-creatinine ratio (ACR), lipid profile including total cholesterol (TC), high-density lipoprotein cholesterol (HDL-C), low-density lipoprotein cholesterol (LDL-C), triglycerides, bilirubin, C-reactive protein, magnesium, calcium, electrolytes (potassium, chloride, sodium), and hepatic cytolysis markers (glutamic oxaloacetic transaminase [GOT], glutamic pyruvic transaminase [GPT], gamma-glutamyl transferase [GGT]). The eGFR was determined using the equation established for the Modification of Diet in Renal Disease (MDRD) Study [13]. The ACR was calculated from spot urine samples as the total albumin divided by creatinine.

Diagnosis of CKD

The classification of CKD based on specific criteria such as eGFR and urinary ACR is crucial for diagnosis and management. CKD was defined as an eGFR less than 60 mL/minute/1.73 m^2^. Microalbuminuria was defined as an ACR between 30-300 mg/g, and macroalbuminuria was established in patients with ACR values exceeding 300 mg/g. Moreover, Sudoscan integrates algorithms that merge electrochemical skin conductance with variables such as body weight, height, age and HbA1c levels. This generates a score known as the Sudoscan-Nephro score, which estimates the current risk of kidney dysfunction.

Diagnosis of CAN

CAN evaluation included ECG, QTc interval assessment, and Cardiovascular Autonomic Reflex Tests (CARTs). CARTs assessed heart rate variability, Valsalva maneuver, orthostatic changes, and blood pressure during an isometric effort. ESP-01-PA Ewing Tester was used. Furthermore, Sudoscan includes embedded algorithms that combine electrochemical skin conductance with age to produce a score predicting the existing risks of CAN (Sudoscan-CAN score).

Sudomotor function assessment

Sudoscan is a device approved by the Food and Drug Administration (FDA) to assess sudomotor function [14]. This is a non-invasive diagnostic test used for assessing sudomotor function, which measures the sweat gland activity primarily in the hands and feet. It involves placing the patient's hands and feet on electrodes for approximately three minutes. The device delivers a small electrical stimulus to stimulate sweat gland activity and then measures the response. This test aids in evaluating the function of the autonomic nervous system and provides valuable information for conditions such as diabetic neuropathy and CKD. Sudoscan also incorporates algorithms that utilize electrochemical skin conductance, along with demographic and clinical factors such as age, height, weight, and HbA1c level, to generate scores indicative of the risk of kidney dysfunction (Sudoscan-Nephro score) and cardiovascular autonomic neuropathy (Sudoscan-CAN score). It is a painless and rapid procedure that requires no preparation.

The statistical analysis of the population involved utilizing IBM SPSS Statistics for Windows, Version 20.0 (Released 2011; IBM Corp., Armonk, NY). Continuous variables that followed a normal distribution were presented as mean ± standard deviation (SD), whereas those not adhering to a normal distribution were expressed as median (interquartile range [IQR]). Categorical variables were conveyed as absolute counts and percentages. Statistical significance was established at a 95% confidence interval (CI). Analysis of variance (ANOVA) was applied to compare groups for quantitative variables, while the χ2 test was employed for categorical variables. Multiple linear regression was conducted to estimate the independent correlation of the Sudoscan-Nephro score with eGFR and albuminuria. The variables that did not follow a normal distribution were log10-transformed to meet the assumption of normality for parametric testing.

## Results

Paraclinical explorations are presented and classified according to the severity of CKD from G1 to G4 (Table 1). The average age increases slightly from group G1 to G3, stabilizing for the more severe disease groups. The systolic blood pressure (SBP) and various risk scores (such as Ewing and Sudoscan scores) vary with the progression of kidney disease. Variables with statistically significant differences (*P* < 0.05) include age, diabetes duration, FPG, urea, uric acid, ACR, Ewing score, and Sudoscan scores, indicating that these significantly change depending on the stage of renal disease. This analysis highlights the importance of continuous monitoring of these indicators in managing the progression of CKD.

**Table 1:** Anthropometric measurements of the groups and laboratory parameters stratified by severity of CKD. ^*^Variables expressed as median, IQR, statistical significance, *P *< 0.05. Data are presented as mean ± SD/median (IQR). BMI, body mass index; SBP, systolic blood pressure; FPG, fasting plasma glucose; HbA1c, glycated hemoglobin; TC, total cholesterol; HDL-C, high-density lipoprotein, LDL-C, low-density lipoprotein cholesterol; TGL, triglyceride; GOT, glutamic oxaloacetic transaminase; GPT, glutamic pyruvic transaminase; GGT, gamma-glutamyl transferase; ACR, albumin-to-creatinine ratio; IQR, interquartile range

CKD	G1 (*n *= 74)		G2 (*n *= 125)		G3a (*n *= 55)		G3b (*n *= 14)		G4 (*n *= 3)		Total		*P*-value
	Mean	Std. deviation	Mean	Std. deviation	Mean	Std. deviation	Mean	Std. deviation	Mean	Std. deviation	Mean	Std. deviation	
Age (years)	58.77	8.54	62.14	9.26	63.93	8.50	62.57	10.05	61.00	7.00	61.59	9.07	0.021
BMI (kg/m²)	32.41	5.83	31.93	5.19	32.80	5.21	31.48	4.31	33.22	6.28	32.23	5.32	0.830
Waist-hip ratio	1.00	0.07	0.99	0.08	0.99	0.08	0.99	0.06	0.95	0.05	0.99	0.07	0.858
SBP (mmHg)	134.55	17.36	132.14	17.91	137.87	19.46	138.86	18.84	135.00	25.98	134.34	18.24	0.314
Ewing score*	3.5	3	3	3	4	2	6	3	3.00	0.00	3	3	0.001
FPG (mg/dL)	177.32	61.46	185.26	85.24	199.41	80.48	209.79	131.99	345.67	153.07	189.01	83.83	0.007
HbA1c (%)	7.97	1.65	8.02	2.01	8.23	1.58	8.20	1.77	10.87	3.38	8.09	1.85	0.107
TC (mg/dL)	190.60	50.86	202.90	57.08	195.96	54.62	203.57	64.19	186.00	28.79	198.01	55.03	0.617
HDL-C (mg/dL)	48.05	13.56	51.87	14.00	51.52	11.60	48.29	12.13	37.67	3.06	50.42	13.36	0.122
TC/HDL-C	4.22	1.76	4.17	1.41	3.90	1.16	4.50	2.19	4.97	0.94	4.15	1.52	0.517
LDL-C (mg/dL)*	102.5	50.75	92	93.6	125.6	62.18	86	60.2	93.50	14.85	103	74.42	0.918
TGL (mg/dL)*	146.5	178.25	163	158	191	97	176	312.5	273	148.85	170	145	0.464
Potassium (mmol/L)	4.17	0.49	3.97	0.60	4.34	0.45	4.45	0.44	5.52	0.31	4.18	0.58	0.000
Sodium (mmol/L)	141.50	4.40	139.11	4.39	134.57	28.09	141.35	4.50	137.50	2.12	138.68	14.68	0.606
Urea (mg/dL)	33.54	12.40	38.84	15.32	45.19	17.00	62.88	21.64	122.80	60.95	40.39	19.00	<0.001
Uric acid (mg/dL)	4.62	1.55	5.55	1.61	5.52	1.66	5.79	1.83	4.51	2.64	5.23	1.66	0.008
GOT (U/L)*	23	21	23	18	20	18.5	24	9.5	39.0	32.5	22	16	0.460
GPT (U/L)*	26.5	17.3	26	19	26	14.8	27	18	48.0	32	26	17	0.256
GGT (U/L)*	34.45	45	34	45	40	52	28	64	60	80	34	45	0.874
ACR (mg/g)*	12.5	31.34	25.59	62.8	21.9	28.5	80.4	230.49	302.95	19	24.5	44.27	0.005
Toronto score	6.5	6	6	5	6	4	8	5	9.00	2	6	5	0.022
Diabetes duration (years)*	7	10	7	7	7.5	8	12	13	13	13	7	8	0.006
Sudoscan-Nephro score	67.72	15.14	66.69	16.24	60.78	15.60	51.79	10.27	61.33	11.02	64.93	15.93	0.002
Sudoscan feet score (uS)	77.14	12.60	78.46	11.62	75.40	14.54	64.18	17.17	74.17	14.18	76.69	13.15	0.003
Sudoscan hand score (uS)	68.77	15.60	70.75	12.61	66.59	16.13	61.07	19.47	77.83	2.52	68.94	14.69	0.076

Differences in various clinical and laboratory parameters were analyzed among patient groups with different levels of albuminuria, from A1 to A3 (Table 2). The groups were defined based on urine albumin levels, reflecting the potential progression of kidney disease. We observed that the average age, lipid profile, and vascular indicators, such as SBP, TC, and HDL-C, did not show significant statistical differences between groups. The duration of diabetes, FPG, Sudoscan scores, and urea showed significant differences (*P *< 0.001) as albuminuria increased. These findings underscored the importance of monitoring albuminuria as an indicator of renal disease progression in the clinical management of patients.

**Table 2:** Anthropometric measurements of the groups and laboratory parameters stratified by severity of albuminuria. *Variables expressed as median, IQR. Statistical significance, *P *< 0.05. Data are presented as mean ± SD/median (IQR). BMI, body mass index; SBP, systolic blood pressure; FPG, fasting plasma glucose; HbA1c, glycated hemoglobin; TC, total cholesterol; HDL-C, high-density lipoprotein, LDL-C, low-density lipoprotein-cholesterol; TGL, triglyceride; GOT, glutamic oxaloacetic transaminase; GPT, glutamic pyruvic transaminase; GGT, gamma-glutamyl transferase; IQR, interquartile range

Albuminuria	A1 (*n *= 176)		A2 (*n *= 83)		A3 (*n *= 12)		Total		*P*-value
	Mean	Std. deviation	Mean	Std. deviation	Mean	Std. deviation	Mean	Std. deviation	
Age (years)	61.38	8.97	62.66	9.37	57.42	7.57	61.59	9.07	0.150
BMI (kg/ m²)	31.99	5.26	32.50	5.32	33.86	6.26	32.23	5.32	0.429
Waist-hip ratio	0.99	0.07	1.00	0.07	1.01	0.09	0.99	0.07	0.354
SBP (mmHg)	133.99	17.96	134.46	19.22	138.67	15.96	134.34	18.24	0.691
Ewing score*	3	2	5	4	6	4	4	4	<0.001
FPG (mg/dL)	176.73	76.09	204.61	82.98	261.17	138.26	189.01	83.83	<0.001
HbA1c (%)	7.84	1.81	8.44	1.78	9.40	2.07	8.09	1.85	0.002
TC (mg/dL)	198.52	51.66	198.45	62.99	187.50	46.27	198.01	55.03	0.796
HDL-C (mg/dL)	50.22	13.63	51.58	13.28	45.28	8.50	50.42	13.36	0.297
TC/HDL-C	4.19	1.50	4.07	1.62	4.20	1.03	4.15	1.52	0.848
LDL-C (mg/dL)*	103.5	54.9	99	88.7	97.80	86.5	103	74.42	0.735
TGL*	153	82.25	211	219	191.5	106.5	170	145	0.606
Potassium (mmol/L)	4.22	0.50	4.07	0.60	4.44	0.94	4.18	0.58	0.245
Sodium (mmol/L)	137.82	19.88	140.01	4.75	137.69	3.61	138.68	14.68	0.797
Urea (mg/dL)	37.44	15.27	41.77	17.70	61.96	38.99	40.39	19.00	<0.001
Uric acid (mg/dL)	5.17	1.67	5.47	1.68	4.42	1.28	5.23	1.66	0.148
GOT (U/L)*	24	17.3	22	15.5	21	16.5	22	16	0.672
GPT (U/L)*	26.5	17.3	25	18	30	21	26	17	0.716
GGT (U/L)*	32	43	37	44	64	78	34	45	0.769
Toronto score*	6	5	7	4	8.5	4	6	5	<0.001
Diabetes duration (years)*	5.5	7	9	7	21	14	7	8	<0.001
Sudoscan-Nephro score	67.14	16.31	60.46	13.52	63.67	20.21	64.93	15.93	0.006
Sudoscan feet score (uS)	79.34	8.96	72.26	17.41	68.54	19.39	76.69	13.15	<0.001
Sudoscan hand score (uS)	69.37	13.84	67.77	15.69	70.88	20.03	68.94	14.69	0.643

The statistical analysis of chronic diabetic complications in patients with CKD across stages G3a, G3b, and G4 showed increasing prevalence rates for diabetic polyneuropathy (DPN) and diabetic retinopathy (DR) with the progression of CKD (Table 3). Specifically, DPN prevalence increased from 69.09% (*n *= 38) in G3a to 92.85% (*n *= 13) in G3b, reaching 100% (*n *= 3) in G4. Similarly, DR showed a prevalence of 52.72% (*n *= 29) in G3a, increasing slightly in G3b (*n *= 8, 57.14%) and reaching 100% (*n *= 3) in G4. Conversely, CAN showed a high prevalence in G3a (*n* = 32, 58.18%) and G3b (*n *= 11, 78.57%), but it is absent in G4. These trends indicated that neurological and retinal complications are strongly correlated with the severity of CKD, emphasizing the importance of early detection and management to mitigate these severe outcomes as CKD progresses.

**Table 3:** The prevalence of chronic complications in patients with T2DM according to the stage of CKD. Data are presented as *n* (%). DPN, diabetic polyneuropathy; DR, diabetic retinopathy; CAN, cardiovascular autonomic neuropathy; T2DM, type 2 diabetes mellitus; CKD, chronic kidney disease

Chronic kidney disease	G3a (*n *= 55)	G3b (*n *= 14)	G4 (*n *= 3)
DPN, *n* (%)	38 (69.9%)	13 (92.85%)	3 (100%)	
CAN, *n* (%)	32 (58.18%)	11 (78.57%)	0	
DR, *n* (%)	29 (52.72%)	8 (57.14%)	3 (100%)	
PAD, *n* (%)	11 (20%)	2 (14.28%)	2 (66.66%)	

Considering the natural progression of CKD, we calculated the risk of progression to dialysis for patients with kidney disease, using KDIGO [15]. Thus, we evaluated anthropometric measurements and laboratory parameters across groups stratified by their risk of progression to dialysis (Table 4). Notably, parameters such as BMI, SBP, diastolic blood pressure, and cholesterol levels did not show significant statistical differences across the risk categories (Table 4). However, variables related to diabetes management and kidney function, such as the duration of diabetes, FPG, urea, eGFR, ACR, Ewing score, Sudoscan-Nephro score, Sudoscan-CAN score, and cardiovascular risk score, demonstrated significant differences (Table 4). These findings highlighted worsening kidney function and elevated risk factors in higher risk groups, illustrating their importance in predicting the risk of dialysis among patients with diabetes.

**Table 4:** Anthropometric measurements of the groups and laboratory parameters stratified by risk of progression to dialysis. *Variables expressed as median, IQR. Statistical significance, *P* < 0.05. Data are presented as mean ± SD/median (IQR). BMI, body mass index; SBP, systolic blood pressure; DBP, diastolic blood pressure; HR, heart rate; FPG, fasting plasma glucose; HbA1c, glycated hemoglobin; eGFR, estimated glomerular filtration rate;  ACR, albumin-to-creatinine ratio; TC, total cholesterol; HDL-C, high-density lipoprotein-cholesterol; LDL-C, low-density lipoprotein-cholesterol; TGL, triglyceride; IQR, interquartile range

Risk of progression to dialysis	Low		Moderately increased		High		Very high		*P*-value
	Mean	Std. deviation	Mean	Std. deviation	Mean	Std. deviation	Mean	Std. deviation	
BMI (kg/m²)	32.05	5.61	32.10	4.92	32.68	4.48	32.96	5.90	0.935
SBP (mmHg)	133.41	17.48	131.91	19.67	141.41	17.99	137.37	18.46	0.143
DBP (mmHg)	76.36	10.52	76.09	10.51	80.72	12.44	73.11	11.12	0.117
HR (bpm)	74.58	10.59	74.42	11.23	77.19	10.85	75.37	8.82	0.654
Diabetes duration (years)*	5	8	8	7	10	5	12	7	<0.001
FPG (mg/dL)*	159	85.5	169	103.5	196	185	176	236	0.01
HbA1c (%)	7.81	1.90	8.34	1.91	8.64	1.47	8.55	2.15	0.071
Potassium (mmol/L)	4.16	0.50	3.96	0.65	4.17	0.52	4.67	0.60	0.024
Urea (mg/dL)	35.61	14.00	39.62	16.70	41.54	13.16	72.66	34.08	<0.001
eGFR (mL/minute/1.73 m^2^)	84.41	1.24	72.63	1.34	60.47	1.35	37.97	1.27	<0.001
ACR (mg/g)	6.44	2.90	31.20	3.30	72.04	3.12	133.16	3.46	<0.001
TC (mg/dL)	198.46	51.35	194.44	63.47	197.91	54.36	201.47	58.68	0.964
HDL-C (mg/dL)	50.36	14.06	51.34	14.31	51.73	10.19	45.95	11.40	0.599
LDL-C (mg/dL)	110.64	46.66	105.36	52.35	111.17	52.88	103.62	51.31	0.924
TGL (mg/dL)*	160	119	180	193.5	192	119	188	135.4	0.509
Ewing score*	3	3	4	3	4	3	4	4	<0.001
Sudoscan-Nephro score (uS)	66.12	1.27	61.42	1.25	57.98	1.31	53.59	1.25	0.01
Sudoscan-CAN score (uS)	31.21	10.10	34.83	9.07	36.72	7.68	39.68	6.40	<0.001
Cardiovascular risk score	26.52	9.18	32.10	12.52	36.99	13.46	48.28	16.83	<0.001

The prevalence of CKD in our group was 26.5% (*n *= 72). Regarding CKD stages, among females, 70.6% (*n *= 101) did not have kidney disease (CKD-), while 21.7% (*n *= 31) were in stage G3a, 6.3% (*n *= 9) in stage G3b, and 1.4% (*n *= 2) in stage G4 of kidney disease (CKD+). Similarly, males exhibited comparable distributions, with 76.6% (*n *= 98) in the CKD- category, 18.7% (*n *= 24) in stage G3a, 3.9% (*n *= 5) in stage G3b, and 0.8% (*n *= 1) in stage G4. Concerning albuminuria, among females, 65% (*n *= 93) fell into the A1 category, 30.8% (*n *= 44) in A2, and 4.2% (*n *= 6) in A3. Males also demonstrated similar patterns, with 64.8% (*n *= 83) in A1, 30.5% (*n *= 39) in A2, and 4.7% (*n *= 6) in A3.

**Figure 1:**
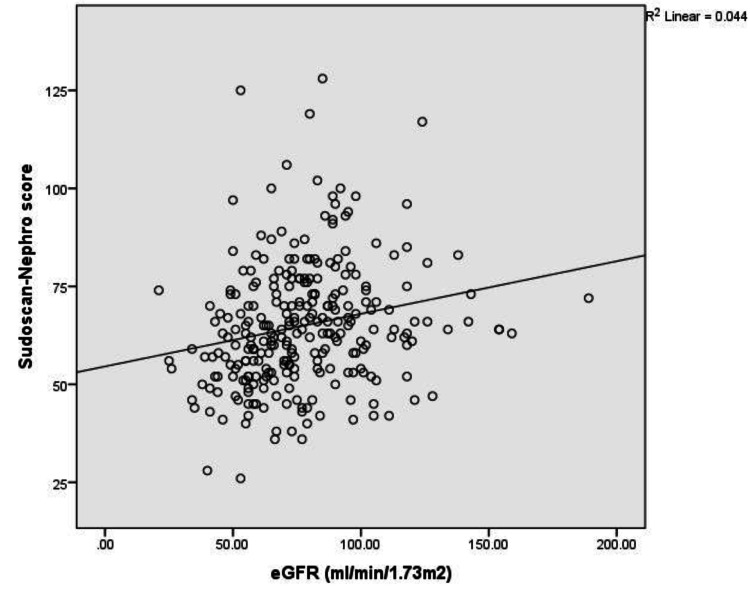
Scatterplot showing the relationship between Sudoscan-Nephro score and eGFR. Diagonal segments are produced by ties. eGFR, estimated glomerular filtration rate; ROC, receiver operating characteristic

The scatterplot showing the relationship between the Sudoscan-Nephro score and the eGFR, with an* R*² value of 0.044, suggested a moderate correlation between these two variables (Figure 1). This relatively substantial correlation implied that with the increase in the Sudoscan-Nephro score, an associated improvement in kidney function was observed, as measured by eGFR. This suggested that the Sudoscan-Nephro score could be a valuable tool for predicting kidney health. However, it is essential to consider other factors and clinical assessments when evaluating kidney function.

**Figure 2:**
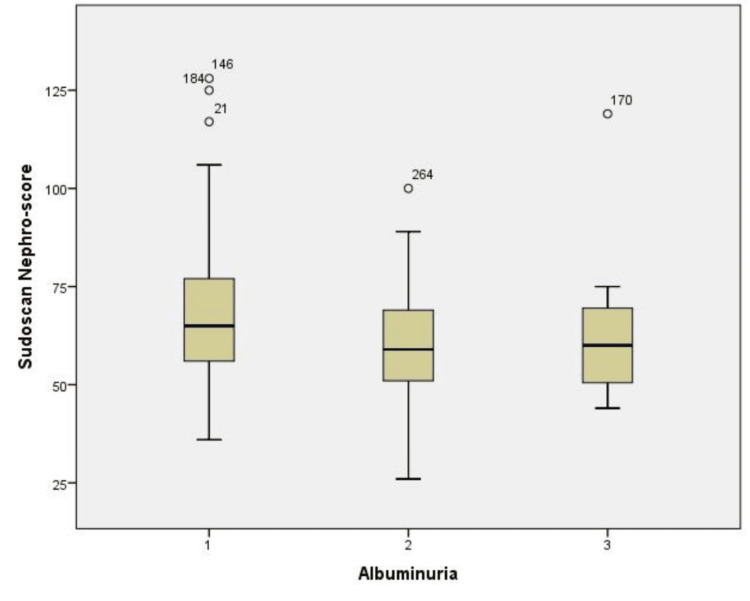
Boxplot showing the relationship between Sudoscan-Nephro score and albuminuria.

This boxplot illustrates the Sudoscan-Nephro scores across three levels of albuminuria (Figure 2). It is observed that there is an inversely proportional relationship between the Sudoscan-Nephro score and the level of albuminuria, with statistical significance (*P *= 0.006).

Performance of Sudoscan in detecting CKD

**Figure 3:**
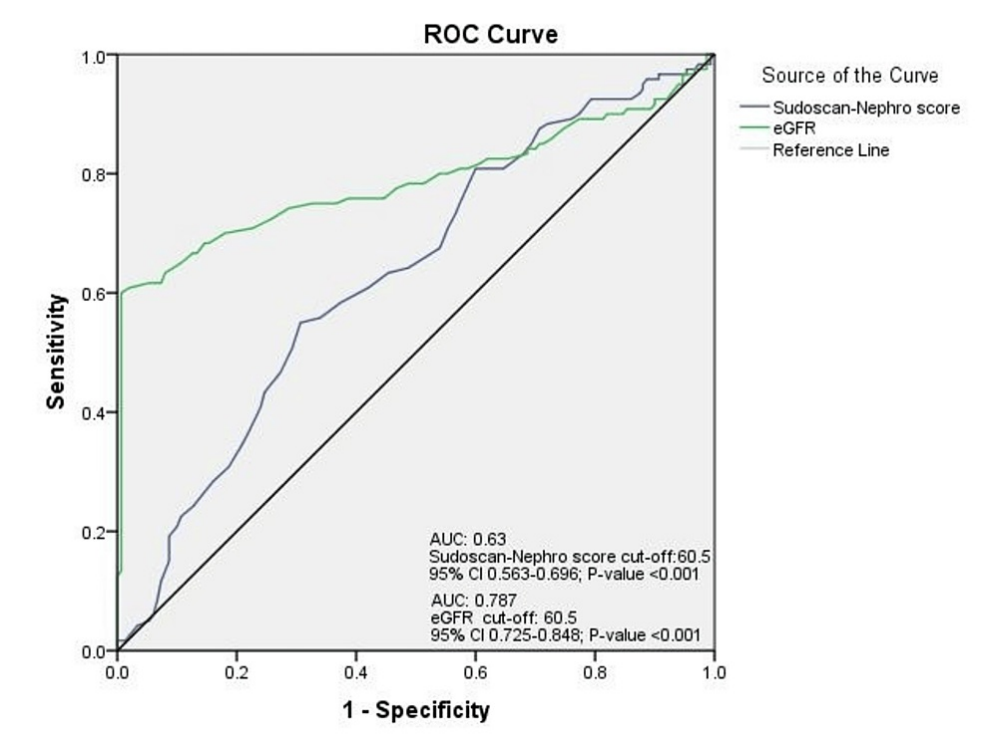
ROC curve of Sudoscan-Nephro score and eGFR in detecting CKD in patients with T2DM. Diagonal segments are produced by ties. Statistical significance, *P *< 0.05. eGFR, estimated glomerular filtration rate; CKD, chronic kidney disease; T2DM, type 2 diabetes mellitus; ROC, receiver operating characteristic

The area under the receiver operating characteristic (ROC) curve of the Sudoscan-Nephro score to predict CKD was 0.63 (95% CI 0.563-0.696). The Sudoscan-Nephro score cutoff was 60.5, and the test had 55% sensitivity and 30.7% specificity to detect CKD. On the other hand, the eGFR (mL/minute/1.73 m^2^) test had a higher area under the curve (AUC) of 0.787 (95% CI 0.725-0.848). The eGFR cutoff was 60.5, and the test had 60% sensitivity and 73% specificity to detect CKD (Figure 3).

**Figure 4:**
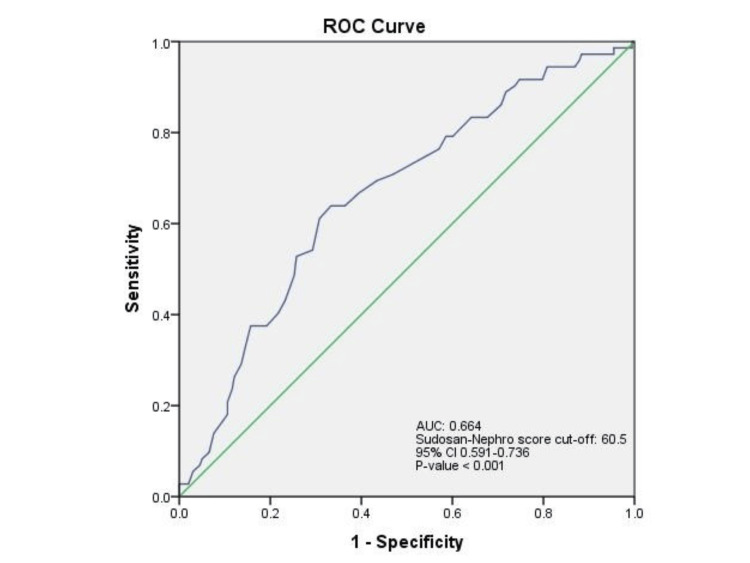
ROC curve of Sudoscan-Nephro score in detecting CKD defined as eGFR <60 mL/minute/1.73 m2. Diagonal segments are produced by ties. Statistical significance, *P* < 0.05. CKD, chronic kidney disease; eGFR, estimated glomerular filtration rate; ROC, receiver operating characteristic

The area under the ROC curve of Sudoscan‑Nephro score to predict CKD (eGFR < 60 mL/minute/1.73 m^2^) was 0.664 (95% CI 0.591-0.736), which is statistically significant (*P *< 0.001). The Sudoscan-Nephro score cutoff was 60.5, and the test had 63.9% sensitivity and 33.3% specificity to detect CKD, defined as eGFR below 60 mL/minute/1.73 m^2^. These attributes highlighted the potential utility of the Sudoscan test as a diagnostic tool, especially in identifying positive cases crucial for clinical management (Figure 4).

**Figure 5:**
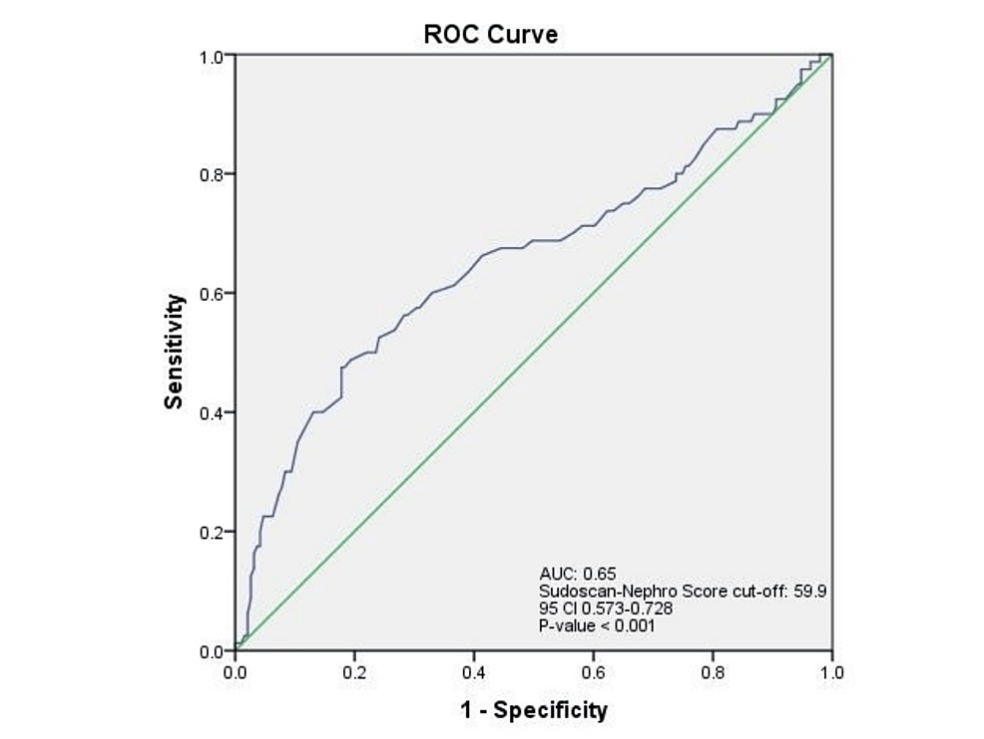
ROC curve of Sudoscan-Nephro score in detecting microalbuminuria. Diagonal segments are produced by ties. Statistical significance, *P* < 0.05. eGFR, estimated glomerular filtration rate; ROC, receiver operating characteristic

The area under the ROC curve of Sudoscan-Nephro score to predict microalbuminuria was 0.65 (95% CI 0.573-0.728, *P *< 0.001). This suggested that the test was moderately effective at distinguishing between individuals with and without microalbuminuria, a key feature for screening tools. The Sudoscan-Nephro score cutoff was 59.9, and the test had 47.5% sensitivity and 17.8% specificity to detect microalbuminuria defined by ACR values between 30 and 300 mg/g (Figure 5).

Table 5 highlights the differences in health indicators between the two analyzed patient groups, stratified by the cutoff value of the Sudoscan-Nephro score in diagnosing CKD, 60.5. The results highlighted that patients with a higher Sudoscan-Nephro score (>60.5) tend to be younger and exhibit better renal function, as evidenced by lower creatinine levels and higher eGFR, compared to those with lower scores. Statistically significant differences were also observed in other parameters such as urea, uric acid, TC, and LDL-C levels (all *P *< 0.05), pointing toward a healthier metabolic and renal profile in the higher-scoring group. Sudoscan-Nephro scores may help assess overall renal and metabolic health in clinical settings.

**Table 5:** Clinical characteristics of patients stratified by Sudoscan-Nephro score cutoff. *Variables expressed as median, IQR. Statistical significance, *P* < 0.05. Data are presented as mean ± SD/median (IQR). BMI, body mass index; SBP, systolic blood pressure; eGFR, estimated glomerular filtration rate; ACR, albumin-to-creatinine ratio; FPG, fasting plasma glucose; HbA1c, glycated hemoglobin; TC, total cholesterol; HDL-C, high-density lipoprotein; LDL-C, low-density lipoprotein; TGL, triglyceride; GOT, glutamic oxaloacetic transaminase; GPT, glutamic pyruvic transaminase; GGT, gamma-glutamyl transferase; IQR, interquartile range

Indicators	Sudoscan-Nephro score				*P*-value
	>60.5 (*n *= 166)		<60.5 (*n *= 105)		
	Mean	Std. deviation	Mean	Std. deviation	
Age (years)	57.96	8.58	66.75	7.05	<0.001
BMI (kg/ m²)	32.05	5.56	32.48	4.98	0.515
Waist-hip ratio	0.99	0.07	0.99	0.08	0.525
SBP (mmHg)	133.07	18.61	136.14	17.61	0.172
Urea (mg/dL)	37.49	15.54	44.61	22.58	0.011
Creatinine (mg/dL)	0.91	0.30	1.05	0.32	<0.001
eGFR (mL/minute/1.73 m²)	83.24	25.30	68.53	21.63	<0.001
ACR (mg/g)*	20.5	33.37	27.07	64.98	0.595
Uric acid (mg/dL)	5.01	1.62	5.54	1.69	0.033
Ewing score*	3	3	4	3	<0.001
FPG (mg/dL)*	180	118.5	169.5	111	0.889
HbA1c (%)	8.13	1.94	8.03	1.72	0.648
TC (mg/dL)	203.86	55.85	189.76	53.01	0.038
HDL-C (mg/dL)	50.79	13.61	49.89	13.05	0.586
CT/HDL-C	4.24	1.54	4.03	1.48	0.284
LDL-C (mg/dL)	114.58	48.76	101.42	48.72	0.032
TGL (mg/dL)*	173	180	171.5	140.75	0.798
Potassium (mmol/L)	4.14	0.42	4.24	0.73	0.428
Sodium (mmol/L)	141.57	3.15	135.19	21.15	0.044
GOT (U/L)*	22	19	22.5	11.3	0.608
GPT (U/L)*	28	16.5	24	14.8	0.597
GGT (U/L)*	34	43	35.5	62	0.608
Toronto score*	6	5	7	4	0.001
Diabetes duration (years)*	6	7	10.5	10	<0.001
Sudoscan feet score (uS)	81.87	8.76	69.35	14.78	<0.001
Sudoscan hand score (uS)	71.61	13.60	65.16	15.40	<0.001

Multiple linear regression revealed several key findings regarding factors influencing Sudoscan-Nephro scores. Older age and longer duration of diabetes are associated with lower Sudoscan-Nephro scores, highlighting the age and disease duration effects on neurological and vascular health. Conversely, a good glucose control, reflected in lower HbA1c levels and higher eGFRs, indicative of normal kidney function, correlated with higher Sudoscan-Nephro scores. Additionally, higher diastolic blood pressure in the supine position positively impacts Sudoscan-Nephro scores. Lower scores on tests such as Ewing and Toronto were significantly associated with lower Sudoscan-Nephro scores, underscoring the importance of neurological and cardiovascular function in determining Sudoscan-Nephro outcomes. These findings collectively emphasized the multifactorial nature of Sudoscan-Nephro scores, with age, diabetes control, kidney function, blood pressure, and specific test scores, all playing crucial roles in assessing screening complications (Table 6).

**Table 6:** Clinical factors associated with Sudoscan-Nephro scores in patients with type 2 diabetes using multiple linear regression. Statistical significance, *P *< 0.05. FPG, fasting plasma glucose; HbA1c, glycated hemoglobin; eGFR, estimated glomerular filtration rate; SBP, systolic blood pressure; DBP, diastolic blood pressure

	Standard β-coefficient	*P*-value
Age	-0.524	<0.001
Diabetes duration	-0.271	<0.001
FPG	-0.155	0.303
HbA1c	0.390	0.005
eGFR	0.183	0.003
Albuminuria	-0.126	0.039
Creatinine	-0.139	0.022
Uric acid	-0.421	0.209
SBP supine position	-0.183	0.007
DBP supine position	0.256	<0.01
Ewing test score	-0.388	<0.001
Toronto test score	-0.264	<0.001

## Discussion

In our study, the prevalence of CKD in patients with T2DM was 26.5%. Our data were similar to the information in the specialized literature, where the prevalence of CKD varied between 24.4% and 27% [16,17].

Hypertension represents a significant risk factor for the development and progression of CKD. Antihypertensive therapy reduces the risk of albuminuria, and in individuals with T2DM and stabilized CKD (eGFR), angiotensin-converting enzyme (ACE) inhibitor or angiotensin II receptor blocker (ARB) therapy reduces the risk of progression to end-stage kidney disease [18-20]. With a non-invasive, cost-effective, and readily deployable method such as Sudoscan, a more detailed understanding of CKD progression in patients with diabetes can be achieved, facilitating early interventions that could delay or prevent the onset of severe renal disease.

The current study highlights the potential of Sudoscan, a non-invasive device that measures skin electrochemical conductance, in detecting CKD in patients with T2DM. The results emphasized a moderate correlation between Sudoscan scores and traditional markers of kidney health, such as eGFR and albuminuria levels. This correlation suggested that Sudoscan could be helpful in assessing early renal dysfunction, an area where traditional methods have limitations in sensitivity and specificity.

The study by Chiu et al. highlighted the potential of Sudoscan to assess sudomotor function in patients with nondialysis CKD across all stages, noting an increased severity in stages 4-5 [21]. The findings revealed that patients with diabetes exhibited more pronounced sudomotor dysfunction compared to non-diabetics, suggesting a complex interaction between diabetes and kidney disease exacerbating neuropathy. Notably, while the correlation between clinical neuropathy scores and sudomotor function implieed that Sudoscan could predict peripheral neuropathy in patients with CKD, eGFR was not identified as an independent risk factor for sudomotor dysfunction, indicating that other factors contributing to uremic neuropathy warrant further investigation. This research underscores the utility of Sudoscan in diagnosing early neuropathic complications in CKD, potentially leading to better-targeted interventions and improved patient outcomes.

In another study on the effectiveness of Sudoscan for screening CKD among patients with T2DM, Mao et al. explored the device's utility in a Chinese cohort from September 2014 to September 2015 [22]. Their research, involving 176 patients, demonstrated that Sudoscan had a sensitivity of 57.8% and a specificity of 100% at a Sudoscan-Nephro score cutoff of 59.5, with an area under the ROC curve of 0.85, closely comparable to 0.84 for eGFR MDRD and 0.77 for eGFR EPI. Patients with a Sudoscan-Nephro score below 59.5 exhibited significantly lower GFR levels, were typically older with longer durations of T2DM, and had higher risks of diabetic complications such as neuropathy and peripheral vascular disease. These findings highlighted Sudoscan's potential as a useful tool for detecting impaired renal function, indicating its value in screening programs within populations affected by T2DM.

Luk et al. discussed the challenges of regular screening for diabetic kidney disease (DKD) in busy and low-resource settings [11]. Their study conducted between 2012 and 2013 involved 2,833 Hong Kong Chinese adults with T2DM. Sudoscan showed a sensitivity of 76.7%, a specificity of 63.4%, and a positive likelihood ratio of 2.1 at a DKD score cutoff of 53, with an area under the ROC curve of 0.75 for detecting CKD. This evidence suggested that Sudoscan could be an effective tool for detecting at-risk patients, especially useful in outreach or resource-limited settings, potentially improving early detection and management of kidney disease in patients with diabetes.

Therefore, our results were consistent with the data in the medical literature. Data analysis has shown that Sudoscan can offer valuable insights into renal functional status in a fast and straightforward manner, potentially enabling early interventions. It is crucial to emphasize that Sudoscan should not serve as a substitute for standard tests but rather as a supplementary tool, enhancing the efficiency and personalization of patient management.

In our study, we also identified a link between the risk of progression to dialysis and CAN, estimated through the Sudoscan-CAN score. This association was recently described in the specialist literature, in a study conducted by Tang et al. [23]. This is significant as dysfunction of the autonomic nervous system could represent a new therapeutic target for developing treatments aimed at preventing the loss of kidney function in diabetes.

An innovative aspect identified in our study is the highlighting of the link between the Sudoscan score and the risk of progression to dialysis in patients with CKD and T2DM. Our analysis demonstrated that a lower Sudoscan score is associated with an increased risk of progression to severe stages of CKD requiring dialysis. This finding underscores the potential of Sudoscan not only as a screening tool for initial renal dysfunction but also as a valuable predictor of disease progression. Therefore, Sudoscan could be used to identify patients who require more aggressive therapeutic interventions to prevent deterioration of renal function, thus providing new insights into the personalized management of high-risk patients. This connection between Sudoscan score and dialysis risk is a significant contribution to the existing literature and opens new possibilities for research into the applicability of Sudoscan in clinical practice.

Limitations

The present study had several limitations. First, the sample size of this cross-sectional cohort was too small to thoroughly examine the correlation between kidney function and all associated clinical characteristics. Second, Sudoscan served only as a screening tool and was not a substitute for blood and urine tests, which are necessary to quantify renal function. Third, the study was conducted in a single hospital.

## Conclusions

The study's findings support the hypothesis that Sudoscan has significant potential in the early detection of renal dysfunction in patients with T2DM, offering a non-invasive alternative to traditional CKD screening methods. Also, the Sudoscan-Nephro score is associated with a higher risk of progression to dialysis, highlighting its potential utility as a predictive biomarker for assessing the risk of dialysis in patients with CKD.

In conclusion, the data suggest that evaluating sudomotor function with Sudoscan offers a viable method for screening kidney dysfunction in Romanian patients with T2DM.
